# Cellular Response of *Sinorhizobium* sp. Strain A2 during Arsenite Oxidation

**DOI:** 10.1264/jsme2.ME15096

**Published:** 2015-10-17

**Authors:** Koh Fukushima, He Huang, Natsuko Hamamura

**Affiliations:** 1 Center for Marine Environmental Studies, Ehime UniversityMatsuyama 790–8577Japan; 2 Department of Biology, Faculty of Sciences, Kyushu UniversityFukuoka 819–0395Japan

**Keywords:** arsenic stress, arsenite oxidase, *aio*, proteome

## Abstract

Arsenic (As) is a widely distributed toxic element in the environment and microorganisms have developed resistance mechanisms in order to tolerate it. The cellular response of the chemoorganotrophic arsenite (As[III])-oxidizing α-*Proteobacteria*, *Sinorhizobium* sp. strain A2, to arsenic was examined in the present study. Several proteins associated with arsenite oxidase and As resistance were shown to be accumulated in the presence of As(III). A shift in central carbon metabolism from the tricarboxylic acid pathway to glyoxylate pathway was also observed in response to oxidative stress. Our results revealed the strategy of the As(III)-oxidizing *Sinorhizobium* strain to mitigate arsenic toxicity and oxidative damage by multiple metabolic adaptations.

Arsenic (As) is an abundant toxic element in the environment, and exists in four oxidation states (-III, 0, III, and V). The most common oxidation states in natural systems are the trivalent (arsenite; As[III]) and pentavalent (arsenate; As[V]) states, which exhibit different mechanisms of toxicity in microorganisms and other biota (16, 27). Arsenate is a phosphate analog and interferes with critical cellular functions by replacing phosphate. Arsenite strongly reacts with sulfhydryl groups in proteins and is considered to be more toxic than As(V). Previous studies reported that As induced oxidative stress by generating reactive oxygen species (ROS), which also cause cellular damage (10, 18, 22).

Microorganisms have developed resistance mechanisms in order to tolerate As, with some microorganisms utilizing As in respiratory metabolism to gain energy for growth (1, 2, 6, 25). The As resistance mechanism in bacteria, the *ars* system, typically involves the reduction of As(V) to As(III) by arsenate reductase (ArsC), and As(III) is then extruded by a membrane ArsB/ACR3 efflux pump. Although the *ars* operon is variably organized, it is widely distributed and conserved across numerous prokaryotic taxa (12, 24). The oxidation of As(III) coupled to the reduction of O_2_ is catalyzed by arsenite oxidase (Aio) and is regarded as a detoxification mechanism in numerous heterotrophic bacteria (25), some of which are also hyper-tolerant of As(III) (*i.e.* 10~20 mM) (3, 5, 8, 13). These Aio-dependent As(III)-oxidizing bacteria have been detected in numerous As-contaminated environments (5, 11, 20), indicating their vital role in controlling the redox cycling of As in such systems.

More detailed investigations on cellular responses among metabolically versatile groups of As(III)-oxidizers need to be conducted in order to obtain a better understand of how As(III)-oxidizing bacteria have evolved to survive in high As environments. In the present study, we performed a proteome analysis of the chemoorganotrophic As(III)-oxidizing α-*Proteobacteria*, *Sinorhizobium* sp. strain A2, in order to elucidate its cellular responses to As. Strain A2 was used in this study due to its ability to efficiently oxidize (*i.e.* ~100 μM h^−1^ per 10^7^ cells) and tolerate a high concentration (10 mM) of As(III). This strain was recently isolated from mine tailings highly contaminated with As and antimony (1.2 and 2.3 g kg^−1^, respectively) (8).

Strain A2 was grown in the absence (control) or presence of 10 mM As(III) (added as NaAsO_2_) with yeast extract (0.002% [wt/vol]) as a carbon source, and As(III) oxidation was monitored as previously described (8). As shown in Fig. 1A, arsenite oxidation was observed after the culture reached the early-stationary phase with a cell density of ~3.0 × 10^7^ cells mL^−1^ and 85% of added As(III) was oxidized to As(V) within 72 h. The growth of strain A2 was not inhibited in the presence of 10 mM As(III), which confirmed its ability to tolerate a high concentration of As(III).

Strain A2 was previously shown to possess the arsenite oxidase gene (*aioA*) (8), which encodes a protein sharing 99.4% amino acid identity with AioA from the facultative chemolithotrophic As(III)-oxidizing α-*Proteobacteria*, *Ancylobacter dichloromethanicus* (1). In order to examine the expression pattern of *aioA* in the strain A2, qRT-PCR was performed using cells grown with or without 10 mM As(III) and harvested after 24, 48, and 120 h (in triplicate). Total RNA was extracted using the RNeasy mini kit (Qiagen, Chatsworth, CA) followed by a DNase treatment as described previously (9). In qRT-PCR, 25 ng of total RNA was reverse-transcribed, then qPCR was performed in triplicate for each RNA sample using the CFX96 real-time PCR detection system (Bio-Rad) as described previously (19). The bacterial and prokaryote universal primers Bact1369-F and Prok1492-R (26) were used to detect the 16S rRNA gene, while *aoxB*1-2F and *aoxB*M2-1R primers were used for the *aioA* gene (21). A qRT-PCR analysis of *aioA* transcripts (Fig. 1B) showed that *aioA* gene expression levels were elevated during the late-log and early-stationary phases (24 and 48 h), concomitant with the oxidation of As(III) observed after 24 h (Fig. 1A). The relative expression of the *aioA* gene markedly decreased after 120 h, ~2 d after most of the added As(III) had already been oxidized to As(V). In the absence of As(III), no substantial *aioA* gene expression was detected throughout the incubation period (up to 120 h). This result contradicted the case of the As(III)-oxidizing *Agrobacterium tumefaciens* str. 5A, in that *aioAB* gene expression was also observed in the absence of As(III) when *A. tumefaciens* str. 5A cells were in the late-log phase and stationary phase (15). These findings suggested that the *aioAB* genes in *A. tumefaciens* str. 5A were regulated not only by As(III) exposure, but also a quorum-sensing-based response as a second regulatory circuit (15). Although *Sinorhizobium* sp. strain A2 is phylogenetically closely related to *Agrobacterium* (95.7% 16S rRNA sequence identity), these two organisms appear to employ distinct regulatory systems for the oxidation of As(III).

In an attempt to gain further insights into the global cellular response of strain A2 to As(III) exposure, a proteome analysis was conducted to identify strongly induced proteins under As(III)-oxidizing conditions. Whole-cell lysates were prepared from cells grown to the early-stationary phase (48 h) in the absence (control) or presence of 10 mM As(III) (the average cell numbers at the time of harvesting were 5.4 × 10^8^ ± 4.8 × 10^7^ and 5.2 × 10^8^ ± 7.6 × 10^6^, respectively), separated by 2D-PAGE gels, and the proteins that were strongly expressed in the presence of As(III) were further analyzed by LC-MS/MS (See Supplemental material for detailed methods).

Among the up-regulated proteins identified in 2D gels (Fig. 2), a large subunit of arsenite oxidase, AioA, was more abundantly expressed in As(III)-oxidizing cells (5.7-fold change) than in the control (Table 1). The absence of AioB, a small subunit containing the Rieske 2Fe-2S cluster, may have been due to the limited numbers of peptides analyzed or limited resolution of the periplasmic proteins often eliminated with cell debris during sample preparation. A previous proteomic study on As(III)-oxidizing *Rhizobium* sp. NT-26 also detected AioA, but not AioB peptides, while *aioA* and *aioB* genes were both expressed in the presence of As(III) (2). Conversely, neither AioA nor AioB peptides were detected by a proteome analysis of the As(III)-oxidizing β-proteobacterial organotroph, *Herminiimonas arsenicoxydans*, and this was likely attributed to the insufficient amount of Aio peptides in these cells (28). This study confirmed, for the first time, the preferential accumulation of AioA within organotrophic As(III)-oxidizing bacteria, as shown with the chemolithoautotrophic As(III)-oxidizing *Rhizobium* sp. NT-26 (2).

Proteomic evidence also indicated the induction of the *ars* operon in As(III)-oxidizing cells. The expression of the *ars* operon was previously shown to be induced by As(III) and As(V) (14), and the *ars* system may also protect cells from the toxicity of As(V) accumulating during the oxidation of As(III). The protein tyrosine phosphatase (TPT) was over-produced (4.4-fold) in cells with As(III) (Table 1). The gene encoding TPT in *Sinorhizobium* spp. (*e.g. S. meliloti* and *S. medicae*) is located within the *ars* operon adjacent to *arsC*, which encodes arsenate reductase (Fig. 3). Although the specific function of TPT in the metabolism of As currently remains unclear, TPT may be expressed in an operon-coordinated manner. The ArsC and ArsH proteins encoded in the *ars* operon were exclusively detected in As(III)-oxidizing cells by a 1D-LC-MS/MS analysis (supplemental methods and Table S1), and the expression of *acr3*, a gene coding for the As(III) efflux pump located within the *ars* operon, as well as *arsC* was also confirmed by RT-PCR (Fig. S1). These results demonstrated the induction of As-specific resistance mechanisms, the Aio and *ars* systems, in As(III)-oxidizing strain A2 cells. Cells under As(III) stress may require additional energy for these resistance mechanisms. Since strain A2 grew indifferently in the presence or absence of As(III) (Fig. 1A), this strain may gain some energy via the oxidation of As(III); however, we previously failed to demonstrate chemolithoautotrophic growth with As(III) (8). Alternatively, cells under As(III) stress may induce proteins involved in energy production because the up-regulation of proteins involved in oxidative phosphorylation has been reported in As(III)-exposed *H. arsenicoxydans* (28).

An isocitrate lyase (ICL) associated with the glyoxylate pathway in central metabolism was induced by 3.5-fold under As(III)-oxidizing conditions (Fig. 2 and Table 1). The induction of ICL was also previously observed in the chemoorganotrophic As(III)-oxidizing *Thiomonas* sp. 3As (4) and *Rhodococcus* strain (13), as well as in superoxide-stressed *E. coli* (23). The toxicity of As is known to involve the generation of ROS (7). ROS disrupt proteins containing Fe-S clusters, such as fumarase and succinate dehydrogenase, in the tricarboxylic acid (TCA) cycle (Fig. 3). Consequently, a carbon metabolic shift to the glyoxylate pathway enables the metabolism of citrate via the modified TCA cycle in order to compensate for the enzymes damaged by ROS (23). ROS is also generated via the oxidation of NADH during aerobic respiration. Since the glyoxylate pathway does not produce NADH, the metabolic shift from the TCA cycle to glyoxylate pathway reduces the amount of NADH produced, and, thus, may reduce oxidative stress (17, 23). The adaptability of central metabolism may be a common mechanism used by organotrophic bacteria to mitigate ROS stress.

Our results indicated that the success of *Sinorhizobium* sp. strain A2 as an efficient As(III)-oxidizer lay in its ability to overcome the deleterious effects of As toxicity by combined strategies: i) As-specific detoxification pathways (*aio* and *ars* systems), and ii) the remodulation of central metabolic pathways to protect against indirect As toxicity and oxidative stress (Fig. 3). Since strain A2 was cultivated from highly contaminated soil with multiple toxic heavy metals (*i.e.* As, antimony, and chromium; 8), metabolic adaptations in order to mitigate oxidative damage may provide physiological advantages for surviving under complex contaminant environments. The *ars* and *aio* operons are carried by a plasmid in the other α-proteobacterial As(III)-oxidizers *Rhizobium* sp. NT26 (2) and *Sinorhizobium* sp. M14 (6), and the plasmid was shown to be responsible for oxidation of As(III) and tolerance of heavy metals (Co, Cd, and Zn) (6). The acquisition of As-oxidizing ability along with *ars* and other heavy metal resistance genes via a plasmid may explain the common As-specific response observed among the phylogenetically related *Rhizobium-Sinorhizobium-Agrobacterium* members of α-*Proteobacteria*. Conversely, other distinct regulatory mechanisms to cope with As toxicity may have developed independently as a result of their adaptation to specific ecophysiological niches.

## Supplementary Material



## Figures and Tables

**Fig. 1:**
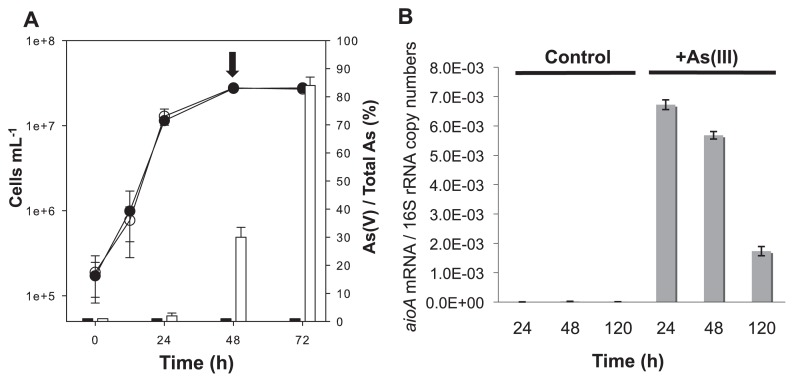
(A) Growth curve and As(III) oxidation profiles of *Sinorhizobium* sp. strain A2 grown in the absence (filled circle) or presence (open circle) of 10 mM As(III). Arsenite oxidation to As(V) is shown in the white column and abiotic control with 10 mM As(III) is shown in the black column as % of As(V) to total As. Each value represents the mean ± standard error of two independent experiments. The arrow indicates the time when cells were harvested for the proteome analysis. (B) Comparison of *aioA* gene expression levels during growth in the absence (control) or presence of As(III). Copy numbers of *aioA* transcripts were normalized by copy numbers of 16S rRNA. Error bars indicate the standard deviation of mean values (*n*=3) from triplicate cultures, with each being obtained from triplicate qPCR technical replicates.

**Fig. 2:**
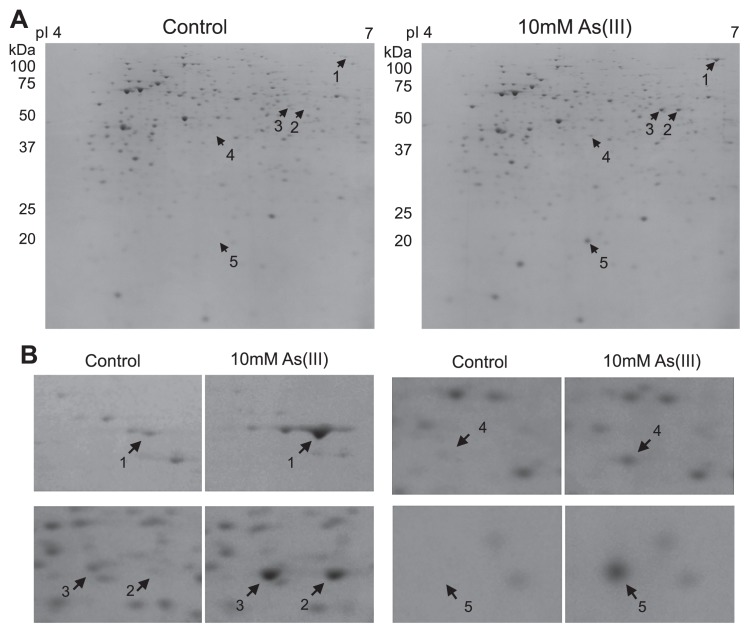
(A) 2D-PAGE proteome maps of proteins differentially expressed in response to As(III). (B) Enlarged images of the protein spots identified in (A). Arrows indicate the proteins accumulated in the presence of As(III), which were further analyzed by LC-MS/MS (Table 1). The number of each spot corresponds to Table 1.

**Fig. 3:**
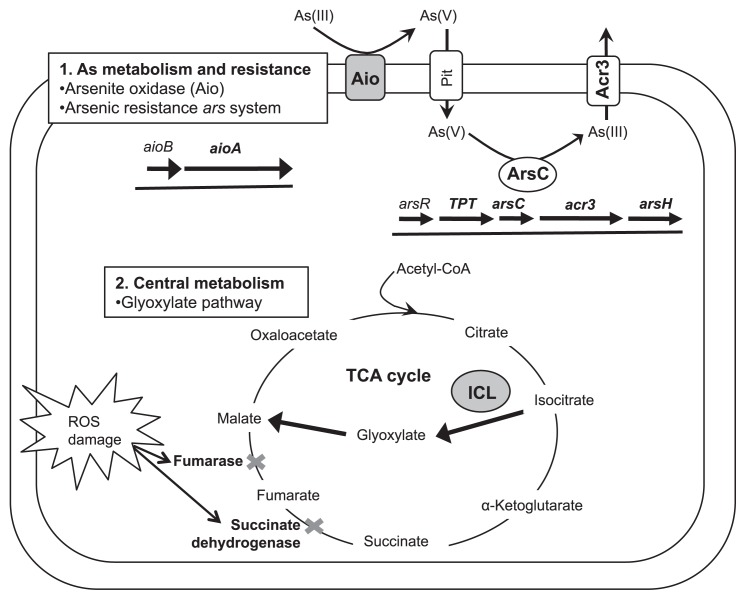
Schematic overview of metabolic adaptation associated with As(III)-oxidation in *Sinorhizobium* sp. strain A2. Proteins and genes encoding proteins that were detected in As(III)-oxidizing cells are shown in a gray background and bold faces, respectively. The physical map of the *ars* operon was adapted from the genome of *Sinorhizobium medicae* WSM419 (NC_009621).

**Table 1:** List of proteins differentially expressed in *Sinorhizobium* sp. strain A2 in the presence of 10 mM As(III)

Spot no.	Protein description	Gene name	NCBI accession no.	Coverage (%)	No. of identified peptides	Fold induction^a^	ANOVA *p*-value^b^
1	Arsenite oxidase large subunit	*aioA*	ADO95186	8.1	8	5.7	0.038
2	Isocitrate lyase	*aceA*	YP_005718903	33.8	16	3.5	0.001
3	Isocitrate lyase	*aceA*	YP_005718903	27.7	6	2.7	0.003
4	Hypothetical protein SMc02703		NP_386471	7.7	2	2.1	0.017
5	Protein tyrosine phosphatase		EHK74782	22.8	5	4.4	0.002

a Fold changes in protein abundances between As(III)-oxidizing cells and control.

b Significance was assessed by a one-way ANOVA.
