# Supplementary material for: Cellular Response of Sinorhizobium sp. Strain A2 during Arsenite Oxidation
Source: Microbes Environ. 2015 Oct 17;30(4):330–4. doi: 10.1264/jsme2.ME15096 (PMC4676556; doi:10.1264/jsme2.ME15096)
Supplement: Supplementary file 1 [file 30_330_s1.pdf]

Supplemental Online Material:

Title: Cellular response of *Sinorhizobium* sp. strain A2 associated with arsenite oxidation

Authors: Koh Fukushima, He Huang, Natsuko Hamamura

Supplemental Methods

Preparation of protein fractions and two-dimensional gel electrophoresis

The cultures were grown in the presence or absence of 10 mM As(III) and cells were harvested after 48 hours by centrifugation (15 min, 8000 × g, 4°C). Average cell numbers at the time of harvesting were $5.2 \times 10^{8} \pm 7.6 \times 10^{6}$ and $5.4 \times 10^{8} \pm 4.8 \times 10^{7}$ for cells grown with and without As(III), respectively. Cell pellets were washed twice with the Xm media and resuspended in the lysis buffer composed of 7 M urea, 2 M thiourea, 3% (w/v) CHAPS, 1% (v/v) Triton X-100, 0.2% (v/v) Bio-Lyte Ampholyte (Bio-Rad, Hercules, CA) and 65 mM dithiothreitol (DTT), followed by sonication on ice. Cell debris were removed by centrifugation (30 min, 20000 × g, 4°C), then the supernatant was recovered, and stored at -80°C. Protein concentration of the extracts was determined spectrophotometrically using the Bio-Rad protein assay reagent (Bio-Rad). The extracts (250 μg) were diluted with the rehydration buffer [7 M urea, 2 M thiourea, 2% (w/v) CHAPS, 0.2% (v/v) Bio-Lyte Ampholyte, 16 mM DTT] and loaded onto the IPG strips (pH 4-7, 17 cm; Bio-Rad). The rehydration of the IPG strip gels was performed in the Protean IEF cell at 50 V at 20°C for 16 h, followed by isoelectric forcusing (linear ramp from 50-250 V for 30 min, linear ramp to 500 V for 30 min, linear ramp to 1000 V for 30 min, linear ramp to 10000 V for 2h, 10000 V for a total focusing time of approximately 55 kVh with a rapid ramp). IPGs were equilibrated with the reducing buffer [6 M urea, 20% (w/v) glycerol, 2% (w/v) SDS, 50 mM Tris-HCl pH 8.8, 1% (w/v) DTT] for 15 min, and the alkylating buffer [6 M urea, 20% (w/v) glycerol, 2% (w/v) SDS, 50 mM Tris-HCl pH 8.8, 2.5% (w/v) iodoacetamide] for 15min. The second dimension was performed with 12% (w/v) SDS-polyacrylamide gels. Proteins were visualized by colloidal Coomassie

G-250 staining and gels were digitized using Chemidoc XRS system (Bio-Rad). Image analysis was performed with the Progenesis SameSpots software (Nonlinear Dynamics, Durham, NC) on the two image sets in duplicate from each of five independent cultures. Spot quantity values were normalized in each gel by dividing the raw quantity of each spot by the total quantity of all the spots included in the gel, and normalized spot values were statistically analyzed by one-way ANOVA.

Sample preparation for nanospray LC-MS/MS analyses

Protein spots exclusively expressed in the presence of As(III) were identified, excised from 2D-gel and destained in 30% (v/v) acetonitrile containing 25 mM NH_4HCO_3. In-gel digestion was performed as previously described (5). Briefly, the gel pieces were reduced in 25 mM NH_4HCO_3 containing 10 mM DTT at 56°C for 60 min, then alkylated in 55 mM iodoacetamide in the dark at room temperature for 45 min, followed by the digestion with 10 ng μl^{-1} trypsin (Promega, Madison, WI) at 37°C overnight. The digested peptides were extracted with the solution consisted of 50% (v/v) acetonitrile and 5% (v/v) arifluoroacetic acid (TFA).

For 1D LC-MS/MS analysis (Table S1), protein extracts were prepared as described above for 2DE gels and precipitated with four volumes of ice-cold acetone at -25°C overnight. Precipitated proteins were separated by centrifugation (10 min, 12000 × g, 4°C), washed twice with ice-cold acetone and homogenized in 50 mM NH_4HCO_3. Protein concentration was determined using the Bio-Rad protein assay reagent (Bio-Rad). The proteins were reduced with DTT at a ratio of 1:20 (w/w) at 56°C for 30 min, alkylated with iodoacetamide at a ratio of 1:4 (w/w) in the dark at room temperature for 30 min. The protein mixture digested with trypsin at a ratio of 1:20 (w/w) at 37°C for 16 h. The digestion was stopped by the addition of 1 µl of formic acid (2). Peptide mixtures were desalted using Sep-Pack C18 cartridge (Waters, Milford, MA) and concentrated to ~100 µl by vacuum centrifugation.

Nanospray liquid chromatography-tandem mass spectrometry (LC-MS/MS) analyses

LC-MS/MS analysis was performed on an LCQ Advantage mass spectrometry system (ThermoFinnigan) coupled to Paradigm MS4 HPLC (Michrom Bioresources) and a Magic C18 column (0.2 × 150 mm, Michrome BioResource). Peptide mixtures (total of 6 μg) were loaded on a Magic C18 column at a flow rate of 1 μl min^{-1}, with a linear gradient from 3–65% of solvent A (2% acetonitrile containing 0.1% formic acid) and solvent B (90% acetonitrile containing 0.1% formic acid) over 300 min. The mass spectrometer was operated in full scan mode (m/z 450-2000). The spray voltage and capillary temperatures were set to 2.5 kV and 200°C. MS/MS spectra were searched using SEQUEST (Bioworks v3.1, ThermoFinnigan) against in-house *Sinorhizobium* database constructed by extracting sequence entries for *Sinorhizoium* spp. from NCBI non-redundant database (as of November 2012). Search parameters were employed from previously reported SEQUEST filtering criteria for validating the identifications (2, 3): $\Delta Cn > 0.1$, *Xcorr* of 1.9, 2.2 and 3.75 for +1, +2 and +3 charge states, respectively. Furthermore, only those proteins with more than two unique peptides or a single unique peptide that has at least seven amino acids and had a minimum of three successive b or y series ions were considered as reliably identified (2, 4).

For 1D LC-MS/MS analysis, semi-quantitative protein abundance was determined as described previously (1, 2) using LC-MS/MS results from triplicate cultures for each treatment. Briefly, the protein abundance index (PAI) was calculated as:

$$PAI=CP/OP$$

where CP is the total peptides count for each detected protein and OP is the number of observable peptides. The OP was calculated by in silico trypsinization of the protein by using the IPEP only proteolysis (http://ipep.moffitt.org/serchProtein.cgi). Protein abundance estimate (emPAI) was then calculated as follows (Table S1).

$$emPAI=10^{PAI}-1$$

References

1. Ishihama, Y., Y. Oda, T. Tabata, T. Sato, T. Nagasu, J. Rappsilber, and M. Mann. 2005. Exponentially modified protein abundance index (emPAI) for estimation of absolute protein amount in proteomics by the number of sequenced peptides per protein. Mol. Cell Proteomics. 4:1265-1272.
2. Luo, Y., X. Ding, L. Xia, F. Huang, W. Li, S. Huang, Y. Tang, and Y. Sun. 2011. Comparative proteomic analysis of *Saccharopolyspora spinosa* SP06081 and PR2 strains reveals the differentially expressed proteins correlated with the increase of spinosad yield. Proteome Sci. 9:40.
3. Peng, J., J. E. Elias, C. C. Thoreen, L. J. Licklider, and S. P. Gygi. 2002. Evaluation of multidimensional chromatography coupled with tandem mass spectrometry (LC/LC-MS/MS) for large-scale protein analysis: the yeast proteome. J. Proteome Res. 2:43-50.
4. Washburn, M. P., D. Wolters, and J. R. Yates III. 2001. Large-scale analysis of the yeast proteome by multidimensional protein identification technology. Nat. Biotech. 19:242-247.
5. Wilm, M., A. Shevchenko, T. Houthaeve, S. Breit, L. Schweigerer, T. Fotsis, and M. Mann. 1996. Femtomole sequencing of proteins from polyacrylamide gels by nano-electrospray mass spectrometry. Nature. 379:466-469.

Supplemental figure S1. Detection of *arsC* and *acr3* transcripts in *Sinorhizobium* sp. strain A2 growing in the presence of 10 mM As(III). Arrows indicate the expected amplification products. Total RNA was prepared from the cells grown with 10 mM As(III) and harvested at 24, 48, and 120 hrs as described in the manuscript for qRT-PCR. RT-PCR was performed using the Access RT-PCR system (Promega, Madison, WI) and the reaction mixture (50 µl) contained 1 µM of each primer and ~50 ng of extracted RNA. Control reactions were performed without addition of reverse transcriptase to verify the absence of DNA in the RNA preparations. Primers for *arsC* (Gene ID: 5319357) and *acr3* (Gene ID: 5319356) were designed based on those sequences from *Sinorhizobium medicae* WSM419 using the Primer3Plus (http://primer3plus.com/cgi-bin/dev/primer3plus.cgi). The primer sets arsC52F (5'-TCGCGGAACACCTTGGCTATG) and arsC384R (5'-TATGAAAGCGCCCTTCTGCTCTTG), and acr755F (5'-TGCCCATCCTCATCCAGGTCTAC) and acr986R (5'-ATCAAGACCACGGACAGCATCAC) were used to amplify *arsC* and *acr3*, respectively.

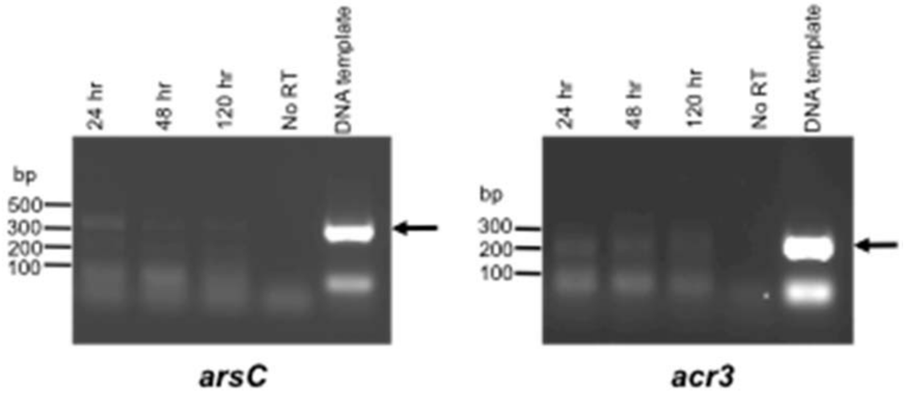

Supplemental table S1. List of arsenic resistance proteins from *Sinorhizobium* sp. strain A2 grown in the presence of As(III) detected by 1D LC-MS/MS.

Functional category	Protein function (gene name)	Accession number	emPAI value[a]	
			+As(III)	Control
Arsenic resistance	Arsenic resistance protein (*arsH*)	YP_001313765	0.55	0.00
	Arsenate reductase (*arsC*) (EC 1.20.4.1)	YP_001313767	1.15	0.00
	Arsenite oxidase, large subunit (*aioA)*	ADO95186	0.42	0.00

[a] emPAI: The exponentially modified protein abundance index value (emPAI) is the transformed ratio of the number of experimentally observed peptides to the total number of peptides obtained after *in silico* trypsinization of the protein calculated as described in supplemental methods.
